# The Impact of Simulated Gastric Acid and Toothbrushing on Surface Characteristics of Resin-Modified Glass-Ionomer Cements

**DOI:** 10.3390/medicina58091149

**Published:** 2022-08-24

**Authors:** Ionuț Tărăboanță, Dan Buhățel, Irina Nica, Simona Stoleriu, Angela Cristina Ghiorghe, Galina Pancu, Andra Claudia Tărăboanță-Gamen, Sorin Andrian

**Affiliations:** 1 Faculty of Dental Medicine, Grigore T. Popa University of Medicine and Pharmacy, 700115 Iasi, Romania; 2 Faculty of Dental Medicine, Iuliu Hațieganu University of Medicine and Pharmacy, 400349 Cluj-Napoca, Romania

**Keywords:** resin-modified glass-ionomer cement, hydrochloric acid, toothbrush, surface roughness

## Abstract

*Background and Objectives*: The aim of this in vitro study was to evaluate the effect of simulated gastric acid associated with toothbrushing on the surface condition of three resin-modified glass-ionomer cements (RMGIC). *Materials and Methods*: One hundred and sixty samples of each material were obtained and included in three study groups according to the tested material: Group I (Ionolux, VOCO GmbH, Cuxhaven, Germany); Group II (Vitremer, 3 M-ESPE, St. Paul, MN, USA); and Group III (Fuji II LC, GC Corporation, Tokyo, Japan). The samples were submersed in hydrochloric acid 0.01 M (pH 3.8) for 3 h and exposed to a toothbrushing procedure at a frequency of 10,000 cycles with medium and hard bristles immediately or 30 min after the acid attack. Profilometric measurements were performed by using a non-contact profilometer (Dektak XT, Bruker, Billerica, MA, USA) in order to assess the surface roughness. ANOVA and Bonferroni post hoc tests were used for the statistical analysis at a significance level of *p* < 0.05. *Results*: Exposure of RMGICs to the erosive effect of hydrochloric acid in association with toothbrushing 30 min after the chemical attack increased the surface roughness of all three RMGICs. Exposure of the three tested materials exclusively to the action of hydrochloric acid did not affect the surface roughness. *Conclusions*: One year of hydrochloric acid challenge associated with one year of toothbrushing with medium-hardness bristles performed 30 min after the acid attack increase the surface roughness of two of the three types of RMGIC tested (Ionolux and Fuji II LC).

## 1. Introduction

With the advent of Bowen resin in the middle of the last century, the era of aesthetic and bioadhesive dental restorations began [1]. Therefore, to the detriment of non-esthetic and non-adhesive materials, composite resins and glass-ionomer cements (GIC) have begun to be widely used. GICs developed by Wilson and Kent [2] have undergone a series of changes over time that have been aimed at improving physical and chemical properties [3]. When compared with composite resins, their physical and mechanical properties are inferior, but they have the advantage of a good and resistant chemical adhesion to dental structures as well as the release of fluoride ions [4,5,6].

To counteract these shortcomings, various strategies were used. So, in the mid-1980s GICs were modified with resins, the resulting materials having superior aesthetic qualities, reduced solubility and setting time and extended working time [3]. Regarding the setting reaction of these materials, an acid-base reaction is associated to photo-polymerization process. Photo-polymerization is possible due to the introduction of hydrophilic monomers such as HEMA (2-hydroxyethylmethacrylate) in a percentage of 4.5% by weight and a photo-initiator [3,7].

Resin-modified glass-ionomer cements (RMGIC) have inferior mechanical and aesthetic properties when compared with composite resins, and the release of fluoride ions is reduced compared with GICs [8].

The longevity of a direct dental restoration depends on the durability and physical properties of the material, such as hardness, wear resistance or surface roughness [9,10]. The surface roughness of restorative materials is an important characteristic because it can increase the surface bacterial colonization [11]. According to a study by Bollen et al. [12], 0.2 μm is the critical value of surface roughness for bacterial retention and adhesion. The erosive wear of restorative materials is caused by extrinsic factors such as acidic food or drinks, lifestyle and environment or by intrinsic factors such as the presence of gastric acid (hydrochloric acid) in the oral cavity in gastroesophageal reflux disease, chronic alcoholism, pregnancy or various psychological disorders such as bulimia or stress rumination [9,13,14]. The prolonged exposure of restorative materials to the action of acids can reduce their mechanical resistance, making them more prone to abrasive wear, such as that produced by the toothbrushing action [9,15]. The exposure of inorganic materials such as GIC to erosive wear causes surface roughness changes, but RMGICs are less prone to this phenomenon [3,16,17].

Toothbrushing is the most widely used oral hygiene technique, but it can affect the surface condition and physical properties of restorative materials by abrasive wear [14]. It has been proved that hydrochloric acid and toothbrushing can act synergistically, having detrimental consequences on the surface condition of resin-based dental materials [4,17].

Therefore, the aim of this in vitro study was to evaluate the erosive effect of hydrochloric acid associated with toothbrushing on the surface roughness of RMGICs. The null hypothesis of the study was that hydrochloric acid in combination with toothbrushing does not have any effect on the surface condition of RMGICs.

## 2. Materials and Methods

**Figure 1:**
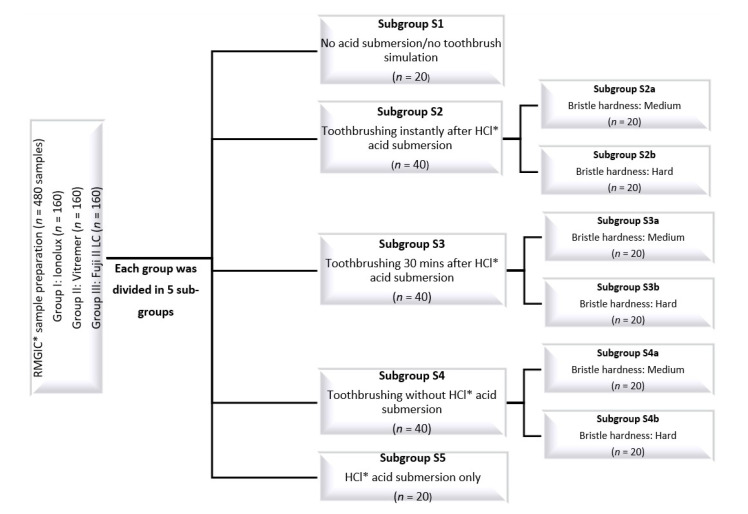
The design of the study. * RMGIC—Resin-modified glass-ionomer cement; HCl—Hydrochloric acid.

The study design is presented in Figure 1.

### 2.1. Sample Size Calculation

In our study, the sample size was determined using G * Power 3.1.9.7 software (Universität Düsseldorf, Germany) with an effect size of 0.3 (medium effect in Cohen classification), an alpha value of 0.05 and 90% power. It was estimated that 19 samples were required for each subgroup.

### 2.2. Sample Preparation

Three different RMGICs were tested in the present study: Ionolux (VOCO GmbH, Cuxhaven, Germany), Vitremer (3 M-ESPE, St. Paul, MN, USA) and Fuji II LC (GC Japan). The detailed composition of each tested material is presented in Table 1. One hundred and sixty samples from each material were caried out and divided into three groups, corresponding to each material used: Group I (Ionolux), Group II (Vitremer) and Group III (Fuji II LC). Cylindrical samples from each material with a 6 mm diameter and 2 mm height were made using an acrylate mold. The mold was placed between two glass plates and a constant pressure with a 500 g weight was applied on the glass plate for 30 s in order to remove the material excess and the air. Celluloid matrices were placed between the glass plates and the mold in order to obtain smooth surfaces. RMGICs were light-cured through the glass plate using an LED light-curing lamp (Woodpecker LED.E, Guilin, Guangxi, China) with a light intensity of 1000 mW/cm^2^ and a wavelength range from 420 to 480 nm. After the samples were removed from the mold, they were stored in distilled water for 24 h.

### 2.3. Finishing and Polishing Procedure

Finishing and polishing procedures were carried out using the Sof-Lex Spiral Finishing and Polishing Wheels System (Batch No. NC11346, 3 M ESPE, St. Paul, MN, USA), composed of two thermoplastic elastomer wheels charged with aluminum oxide particles. The wheels were colored in beige and white according to their abrasiveness. The beige spiral wheel was used for finishing and removal of scratches, and the white wheel was used for the final polishing. Each wheel was used for 30 s only one time for each sample and was activated by a contra-angle handpiece at a speed of 20,000 rpm. By the end of this procedure, 100 samples from each group were subjected to acid submersion (subgroups S2, S3 and S5), 40 samples were directly submitted to toothbrushing simulation (subgroup S4) and 20 samples remained as control (subgroup S1).

### 2.4. Acid Attack Simulation

A hundred samples from each study group were submitted to acid attack simulation by submersion in a hydrochloric acid solution 0.01 M with a pH of 3.8 in a single cycle of 3 h. The pH of the solution was tested using a pH-meter (Thermo Scientific Eutech pH 5+, Vernon Hills, IL, USA). The acid attack simulation was performed at a constant temperature of 37 °C in an incubator (Biobase BJPXH30II, Biodusty, Jinan, China). After this procedure, the samples were stored in distilled water for 24 h. From the total number of the samples, 80 specimens were then submitted to toothbrushing simulation (subgroups S2 and S3), while 20 specimens were not exposed to toothbrushing (subgroup S5).

### 2.5. Toothbrushing Simulation

A total number of 120 samples were subjected to the toothbrushing procedure as follow: 40 samples were brushed immediately after the acid submersion (subgroup S2), 40 samples were brushed 30 min after the acid submersion (subgroup S3) and 40 samples were submitted to toothbrushing only (subgroup S4). The toothbrushing simulation procedure was performed using a brushing machine with a frequency of 100 cycles/min, an intensity of 10,000 brushing cycles and a constant load of 500 g. Half of the samples (subgroups S2a, S3a and S4a) were brushed with toothbrush with medium-hardness bristles (Toothbrush R.O.C.S. Professional Medium, Tallinn, Estonia) made of nylon with a bristle length of 0.8/1.3 cm and thickness of 0.18/0.2 mm. The other half of the samples (subgroup S2b, S3b and S4b) were brushed with a toothbrush with hard bristles (Toothbrush R.O.C.S. Professional Firm, Tallinn, Estonia) made of nylon with the bristle length and thickness not provided by the manufacturer. The toothbrushing procedure was associated with the use of a toothpaste slurry obtained by combining a medium RDA (Relative Dentin Abrasivity) toothpaste (Sensodyne Fresh Mint, GSK, Middlesex, UK) and distilled water in a 1:3 ratio. The samples were then rinsed under running water for 2 min and dried by using the air spray from the dental unit for 2 min.

### 2.6. Surface Roughness Evaluation

The surface roughness evaluation was performed by profilometric measurements, using a non-contact profilometer (Dektak, Bruker, Billerica, MA, USA) and Bruker Software (Bruker, Billerica, MA, USA). For each sample, the profilometric profile and the arithmetic mean roughness values (Ra) were recorded. The value of the cut-off was 0.4 mm, and the navigating distance of the stylus was 4 mm. The tip of the stylus was 5 μm, and it was activated with a force of 4 mN and a speed of 0.5 m/s. Ten measurements with crossing directions were performed for each sample. The mean Ra value was expressed as a result of three distinct determinations, each sample being rotated with a 120° angle.

### 2.7. Statistical Analysis

The data between or within the study groups and subgroups were compared using IBM SPSS 26.0 software. Two-way ANOVA and *post hoc* Bonferonni parametrical tests were used to establish the differences between the mean Ra values with a level of significance of *p* < 0.05.

## 3. Results

**Figure 2:**
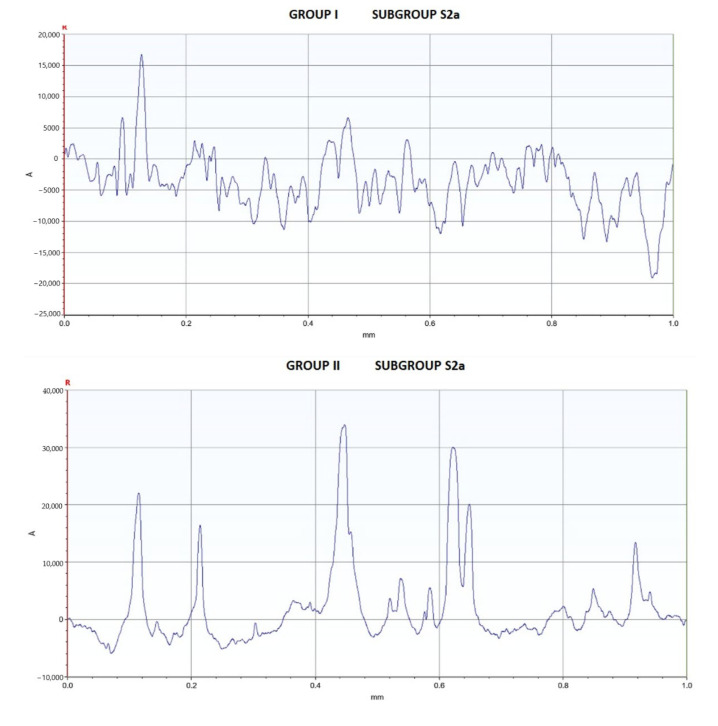

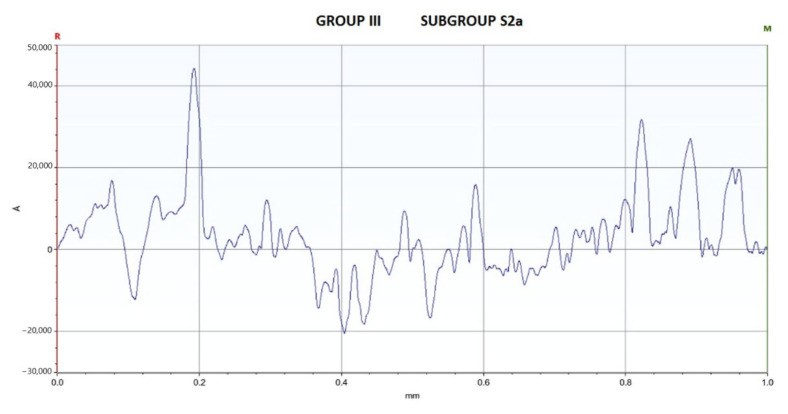
Profilometric measurements of samples in Group I, II and III, subgroup S2a.

**Figure 3:**
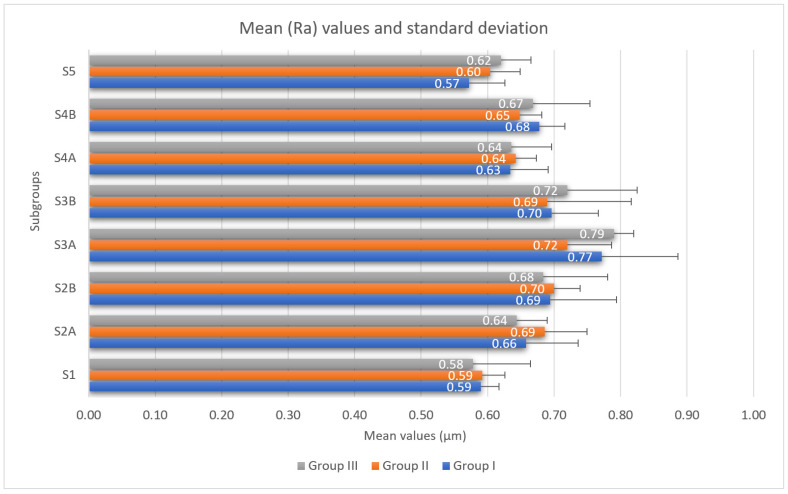
Mean (Ra) values and standard deviation for each subgroup.

Some of the profilometric measurements of the samples in groups I, II and III, subgroup S2a, are shown in Figure 2. The mean Ra values and standard deviation of the surface roughness of each group and subgroup are presented in Figure 3. No differences with statistical significance were found between groups I, II and III in each of the subgroups at a significance level of 0.05.

When analyzing the mean ranks of each subgroup within each group, it can be observed that the highest mean value (Ra) was reached by subgroup S3a for each study group: Group I (0.77 µm); Group II (0.72 µm); and Group III (0.79 µm). The lowest values were recorded by subgroup S5 for group I (0.57 µm) and subgroup S1 for groups II (0.59 µm) and III (0.58 µm).

When comparing the data between the subgroups in each group, statistically significant differences were found in Group I between subgroups S1 and S3a (*p* = 0.034) and subgroups S3a and S5 (*p* = 0.02). In Group III, significant differences between subgroups S1 and S3a (*p* = 0.006) were recorded, and in Group II, no statistically significant differences were found.

## 4. Discussion

Dental materials used for direct restoration are continuously prone to chemical and mechanical aggressions in the oral environment [9]. In our in vitro study, we could not fully mimic the oral environment, but the results obtained may offer a perspective on RMGIC resistance to chemical and physical aggression. The changes in surface condition of RMGICs following exposure to the acidic action of hydrochloric acid and the abrasive effect of toothbrushing was quantified using profilometric measurements, in agreement with other studies that used the same technique [9,18]. The pH of hydrochloric acid used in the study was 3.8, in accord with the results of previous studies that analyzed the gastric contents of patients suffering from bulimia [19,20]. In order to keep the H^+^ ions in a constant concentration, the solution was changed every hour. According to a protocol for simulating the acid attack described by Yehia et al., the immersion time in hydrochloric acid of three hours is equivalent to the exposure to gastric juice for one year [20].

GICs are generally indicated for the treatment of cervical wear [21,22]. This material has the advantage of being adhesive to hard dental tissues and of releasing fluoride ions [21,22,23]. RMGICs have additional advantages when compared with GICs, but the resistance of these materials to mechanical or chemical factors has not been sufficiently assessed [18,24].

RMGIC could have a lower resistance to erosive wear due to the presence of HEMA monomers with a low hardness and a greater predisposition to water absorption when compared with GICs [18,25,26]. The polymerization reaction of RMGIC is performed by HEMA and methacrylate groups together with the polyacrylic acid with which it forms chemical bonds, so the poly-HEMA and polyacrylate salts together create a homogeneous matrix [18]. It has been shown that GIC benefits from a secondary polymerization process [25]. As a result, a hydrated silicate structure is formed, which considerably increases the hardness of the material [24]. In RMGIC, due to the resin matrix that forms the “snap set” phenomenon, the formation of this structure is no longer performed, and this may be a cause of the low strength of the material [2,18].

El-Badrawy et al. suggested that the degradation of materials based on glass-ionomers is due to the leakage of cations under acidic conditions [27]. This fact was explained by Fukazawa et al. [28], who considered that when the material is immersed in acid, the surface of the cement and the matrix gel are infiltrated by the solution, and hydrogen ions (H^+^) lose their place, which is taken up by metal cations (Ca^2+^ or Al^3+^) [11,20]. Subsequently, the free metal cations are released into the solution as a result of the concentration gradient of the solution [20].

The results of a previous personal study conducted on other types of resin-based materials [17,29] showed a similar behavior after their exposure to acid attack with hydrochloric acid. A reason could be the softening or leaching of the monomers from the organic matrix [9,30]. Regarding RMGIC, the change in surface condition is due to the dissolution of the silicon hydrogel layer and the subsequent displacement of the glass particles present in each of the three tested RMGICs (Ionolux, Vitremer and Fuji II LC) [9,18,26]. However, Briso et al. suggested that the products of chemical degradation phenomenon have the ability to increase the pH value of the solution, thus having a beneficial but limited effect against the occurrence of dental erosion [9]. At the same time, the period of action of the acid on the materials is very important [9,20].

The device used in this study to simulate toothbrushing was similar to that developed by Harrington et al. in 1982 [31]. Samples were subjected to 10,000 brushing cycles, equivalent to one year of toothbrushing according to Goldstein and Lerner [32]. Previous studies have shown that brushing without toothpaste does not cause significant loss of substance of restorative materials [18,33,34,35], so that in our study we used a toothpaste (Sensodyne Fresh Mint) with a medium RDA value. In this situation, the abrasion phenomenon can be explained by the interposing of an abrasive body between two other bodies in motion, known as “three-body wear” [18,35]. In our study, it can be observed that one year of hydrochloric acid challenge associated with one year of toothbrushing with medium-hardness bristles performed 30 min after the acid attack increase the surface roughness for two of the three tested materials (Ionolux and Fuji II LC). No changes in surface roughness were found between the three studied materials. No significant increases in the surface roughness were observed for the toothbrushing technique performed with hard bristles. These results are in accordance with a study conducted by Carvalho et al., which showed that the smoother the bristles, the rougher the material surface will be. This conclusion can be explained due to the capacity of the bristles to hold the toothpaste better and the increased flexibility of the filaments that produce a wider contact area between the bristle and the tooth/material surface [36].

The results of previous studies in the literature show contradictory results, some concluding that the degree of abrasion resistance of RMGICs is higher compared with conventional GICs [18,37] and others showing its opposite [2,25]. This may be due to the powder–liquid ratio or to the differences in composition and structure of the poly(acrylic) matrix [9,18]. At the same time, the surface roughness of the restoration materials is changed by a combination of factors such as the powder–liquid ratio, the type of finishing and polishing tools and hydrochloric acid pH and concentration, as well as speed, pressure, type of toothbrush or toothpaste with which the brushing procedure was performed [33]. The surface condition of RMGIC can also be influenced by the incorporation of air bubbles during the mixing of the material, which increases the porosity and implicitly the surface roughness [33,34].

In our study, the RMGIC surface roughness was analyzed by the end of the finishing and polishing procedure, after immersion in hydrochloric acid and after the brushing procedure with medium and hard toothbrush bristles. We observed that the mean Ra values of the surface roughness after each procedure were higher than 0.57 μm, which indicates an increased predisposition to the bacterial plaque retention of these materials. According to the conclusions of Bollen et al., the value of 0.2 μm is the critical one for bacterial adhesion [12]. Momoi et al [18] concluded that all materials that require mixing a powder and a liquid have a higher surface roughness compared with materials presented as a single paste [33]. A possible motivation for the high values of the surface roughness of RMGIC could be the small amount of glass particles that are easily dislocated from the matrix and the increase in the exposure potential of the air bubbles incorporated during mixture [33,34,35,37]. Although RMGIC releases fluoride ions, the loss of surface integrity of the restorations can facilitate the accumulation and development of bacterial biofilm, increasing the risk of secondary caries [28,33,38].

In this study, we analyzed only a few clinical and technological aspects of RMGICs that cannot clearly draw conclusions on the clinical performance of these materials. Additional studies are needed to replicate as accurately as possible the conditions in the oral environment, such as salivary flow, composition of saliva, development of micro-organisms, thermal fluctuation, or enzymes activity. Moreover, future in vivo studies are required in order to validate the results.

## 5. Conclusions

One year of hydrochloric acid challenge associated with one year of toothbrushing with medium-hardness bristles performed 30 min after the acid attack increases the surface roughness of two of the three types of RMGIC tested (Ionolux and Fuji II LC). Exposure of the three tested materials exclusively to the action of hydrochloric acid did not modify the surface roughness. Toothbrushing with medium or hard bristles immediately after the acid attack or without submersion of the samples in hydrochloric acid did not change the surface condition for any of the tested materials.

## Figures and Tables

**Table 1:** The composition of the tested RMGICs.

The Name of RMGIC	Manufacturer	Batch No.	Material Composition
Ionolux	VOCO GmbH, Cuxhaven, Germany	2010139	Liquid: Bis-GMA *, Polyacrilic acid, UDMA *, HEMA *
			Powder: fluoro-alumino-silicate glass
Vitremer	3 M-ESPE, St. Paul, MN, USA	NC32872	Liquid: Polyacrilic acid copolymer, water, HEMA *, carboxylic acid copolymer
			Powder: fluoro-alumino-silicate glass, potassium persulfate, ascorbic acid
Fuji II LC	GC Japan	1911262	Liquid: Acrylic maleic acid copolymer, HEMA *, UDMA *, camphoroquinone
			Powder: fluoro-alumino-silicate glass

* Bis-GMA—bisphenol A diglycidyl ether methacrylate; UDMA—urethane dimethacrylate; HEMA—2-hydroxyethylmethacrylate.

## Data Availability

Not applicable.
